# Neuroprotective effects of maternal melatonin administration in early-onset placental insufficiency and fetal growth restriction

**DOI:** 10.1038/s41390-024-03027-4

**Published:** 2024-01-15

**Authors:** Atul Malhotra, Anna K. A. A. Rocha, Tamara Yawno, Amy E. Sutherland, Beth J. Allison, Ilias Nitsos, Yen Pham, Graham Jenkin, Margie Castillo-Melendez, Suzanne L. Miller

**Affiliations:** 1 The Ritchie Centre, Hudson Institute of Medical Research, Melbourne, VIC Australia; 2 Monash Newborn, Monash Children’s Hospital, Melbourne, VIC Australia; 3 Department of Paediatrics, Monash University, Melbourne, VIC Australia; 4 Department of Obstetrics and Gynaecology, Monash University, Melbourne, VIC Australia

## Abstract

**Background:**

Early-onset fetal growth restriction (FGR) is associated with adverse outcomes. We hypothesised that maternal melatonin administration will improve fetal brain structure in FGR.

**Methods:**

Surgery was performed on twin-bearing ewes at 88 days (0.6 gestation), and FGR induced in one twin via single umbilical artery ligation. Melatonin was administered intravenously (6 mg/day) to a group of ewes commencing on day of surgery until 127 days (0.85 gestation), when the ewe/fetuses were euthanized, and fetal brains collected.

**Results:**

Study groups were control (*n* = 5), FGR (*n* = 5), control+melatonin (control+MLT; *n* = 6) and FGR+melatonin (FGR + MLT; *n* = 6). Melatonin administration did not significantly alter fetal body or brain weights. Myelin (CNPase+) fibre density was reduced in FGR vs. control animals in most brain regions examined (*p* < 0.05) and melatonin treatment restored CNPase fibre density. Similar but less pronounced effect was seen with mature myelin (MBP+) staining. Significant differences in activated microglia (Iba-1) activity were seen between lamb groups (MLT mitigated FGR effect) in periventricular white matter, subventricular zone and external capsule (*p* < 0.05). Similar effects were seen in astrogliosis (GFAP) in intragyral white matter and cortex.

**Conclusions:**

Maternal melatonin administration in early onset FGR led to improved myelination of white matter brain regions, possibly mediated by decreased inflammation.

**Impact:**

- Maternal melatonin administration might lead to neuroprotection in the growth-restricted fetus, possibly via dampening neuroinflammation and enhancing myelination.
- This preclinical study adds to the body of work on this topic, and informs clinical translation.
- Neuroprotection likely to improve long-term outcomes of this vulnerable infant group.

## Introduction

Fetal growth restriction (FGR) is a serious pregnancy complication that most often results from placental insufficiency.^1^ FGR is associated with perinatal mortality and morbidity, and increased risk of neurological deficits in the offspring, including motor, behavioural and cognitive deficits.^2,3^ The timing of onset of placental insufficiency plays a critical role in the risk of neurodevelopmental outcomes of children born growth restricted. Early-onset FGR is arbitrarily set as diagnosis at or before 32 weeks of pregnancy, and late-onset FGR is that diagnosed after 32 weeks, with a 10-fold greater prevalence of late-onset FGR compared to early-onset.^4,5^ Both early and late-onset FGR are linked to significant morbidities, but early-onset FGR is the more severe form, associated with severe placental dysfunction, fetal hypoxia, pre-eclampsia, and patterns of deterioration which progress from escalating Doppler abnormalities to abnormal biophysical parameters, and higher risk of adverse neurodevelopment.^4,5^ Early-onset FGR is more likely to be diagnosed antenatally than late onset,^5^ and therefore may be amenable to antenatal treatment.

In human infants, FGR is strongly associated with altered brain development including a decrease in total brain volume, cortical grey matter volume, cerebellar and hippocampal volumes, reduced or delayed brain myelination, neuronal degeneration, and suboptimal neuronal connectivity.^2,6^ Neurodevelopmental delays are observed in infants and children subsequent to both early- and late-onset FGR, but early-onset FGR results in greater disability, particularly when brain sparing is present.^7^ Few studies have compared neuropathology following early- and late-onset FGR, with the exception of our previous fetal sheep study that showed a greater degree and more widespread white matter brain injury in early-onset FGR that was accompanied by neuroinflammation, compared with late-onset FGR.^8^ Importantly, in both preclinical studies and clinical neuroimaging studies of FGR, neuropathology is already evident in the fetal brain in utero^8–10^ and thus an optimised neuroprotective approach to decrease brain injury in the growth-restricted fetus would ideally be applied before birth. While preclinical and clinical data demonstrate that early-onset FGR presents a more significant challenge and greater degree of brain injury than late-onset FGR, preclinical studies that have specifically examined neuroprotective therapies during pregnancy for early-onset FGR are lacking.

The pineal hormone, melatonin has received strong interest for its neuroprotective benefits, including for the fetal and neonatal brain.^11,12^ It is particularly appealing as a neuroprotective agent for the developing brain because it can be given to the mother during pregnancy^13^ with no deleterious effects, and its small size and amphiphilic nature mean that it can cross the placenta and also the fetal blood brain barrier to act as a neuroprotectant directly to the fetus.^14–16^ A number of studies in small and large animal models of perinatal brain injury have characterised the neuroprotective benefits of melatonin, and shown to be mediated through antioxidant, anti-inflammatory, and anti-apoptotic effects, with additional protection of cerebrovascular structure and blood–brain barrier integrity.^16–20^ Many of the actions of melatonin are mediated through the melatonin receptors, MT1–MT3; however anti-inflammatory and antioxidant effects are not necessarily receptor-mediated.^21^ A handful of preclinical animal studies have also examined whether melatonin has neuroprotective benefits for the developing brain in cases of FGR. These studies reiterate that melatonin protects against a chronic adverse intrauterine environment to normalise brain development.^17,19,22^ To date, however, these studies have assessed antenatal melatonin therapy only in the context of late-onset FGR.^17,19^

Accordingly, in this study we investigated the effects of maternal administration of melatonin on fetal brain structures in early-onset FGR, and assessed presence and distribution of melatonin receptors, MT1 and MT2, in the fetal brain. We hypothesised that antenatal melatonin given to the pregnant ewe carrying an early-onset FGR fetus would mitigate FGR related brain injury seen in the developing brain.

## Materials and methods

### Experimental design

The surgical and experimental procedures undertaken in this project were approved by the Monash Medical Centre (A) Animal Ethics Committee (MMCA2014/04) in accordance with guidelines for animal research set out by the National Health and Medical Research Council of Australia and informed by the international ARRIVE guidelines for reporting of animal research.

Surgery was performed on twin-bearing Border-Leicester-cross Merino crossbred pregnant ewes. Placental insufficiency and subsequent FGR was induced via a surgical procedure in fetal sheep at 88–90 days gestation (equivalent to 0.6 gestation; early onset) where term is ~148–150 days in this cohort of sheep.^8^ Prior to the induction of anaesthesia, all ewes received 1 g ampicillin (Austrapen; Lennon Healthcare, St Leonards, NSW, Australia) and 500 mg engemycin (Coopers, Bendigo East, VIC, Australia) intravenously (i.v.). Anaesthesia was induced with i.v. 20 mg/kg sodium thiopentone (Pentothal; Bomac Laboratories Ltd., Auckland, New Zealand), the ewe was intubated and anaesthesia was maintained with 0–2% isoflurane (Isoflo; Abbott Australasia, Botany, NSW, Australia) in oxygen and air. Under aseptic conditions, the uterus was located via midline abdominal incision and palpated to locate each fetus. The tail end of each fetus was exteriorised and a sterile polyvinyl catheter inserted into the femoral artery of each fetus (ID 0.5 mm, OD 1.0 mm; Critchley Electrical, Kingsgrove, NSW, Australia). FGR was induced in the second twin by placing two silk ligatures tightly around one of the umbilical arteries to fully occlude the artery, termed single umbilical artery ligation (SUAL). The SUAL procedure causes placental insufficiency by inducing necrosis of approximately half of the placental cotyledons supplying that fetus and available for oxygen and nutrient transfer, leading to an increasing degree of fetal hypoxia and subsequent growth restriction.^8,23^ The fetuses were returned to the uterus and the uterine and abdominal incisions were repaired in layers. The ewe was recovered from surgery and received daily antibiotics/ analgesia (paracetamol) for 3 days post-surgery (as above). Fetal arterial blood samples were collected every day for assessment of blood parameters using an ABL 700 blood gas analyser (Radiometer, Copenhagen, Denmark).

Melatonin was administered to a group of ewes carrying a SUAL and control fetus. Melatonin (Sigma-Aldrich, Castle Hill, NSW, Australia) was dissolved in absolute ethanol and diluted to 15 mg melatonin/95 mL using sterile 0.9% saline, so that the concentration of ethanol in the final solution was 1%. At 4 h after SUAL surgery, melatonin-treated ewes received a 1 mg melatonin bolus in 5 mL saline (i.v.) and were then placed on a continuous melatonin infusion of 0.25 mg/3.16 mL/h, thereby delivering 6 mg melatonin every 24 h (equivalent to 0.1 mg/kg based on average ewe weights in mid gestation); this is below the melatonin dosage of >20 mg/kg considered to be anaesthetic. The continuous infusion was delivered with a CADD infusion pump with a 100 mL reservoir (Smiths Medical, St Paul, MN). Each day, the melatonin reservoir was refilled and this was continued for the remainder of the pregnancy (until euthanasia at 125–127 days gestation). The pump was contained in a canvas bag secured on the ewe’s back and the infusion line was covered in black tape to prevent light exposure. Ewes were kept in animal house facilities maintained at an ambient temperature of 20 °C.

At 125–127 days gestational age, the ewe and fetuses were humanely killed via overdose of pentobarbitone (i.v., 100 mg/kg, Virbac Pty Ltd, Australia) to the ewe. The fetuses were removed, weighed and brains collected for analysis as described below.

### Animal groups

Our experimental design resulted in four study groups: control (*n* = 5), FGR (*n* = 5), control lambs administered melatonin (control+MLT; *n* = 6) and FGR lambs administered melatonin (FGR + MLT; *n* = 6).

### Brain pathology

The brain of each fetus was removed, weighed and divided at the midline into right and left hemispheres. The right hemisphere of the brain was further sectioned coronally into 0.5 cm blocks and fixed by immersion in 10% buffered formalin (ProSci Tech, Thuringowa, QLD, Australia) for 3 days, prior to embedding in paraffin. Subsequently, 10 μm coronal sections were cut for analyses. The left half of the brain was also sectioned into 0.5 cm blocks and each block snap frozen for use in a different study.

### Immunohistochemistry

White matter abnormalities were determined using specific markers for oligodendrocyte lineage cells (oligodendrocyte transcription factor 2, Olig-2) and density of myelinated axons (cyclic nucleotide phosphodiesterase, CNPase and myelin basic protein, MBP). Grey matter abnormalities were determined by assessing neuronal integrity (NeuN), while inflammation was determined using an astrocytic (glial fibrillary acidic protein, GFAP) and microglial marker, ionised calcium-binding adaptor molecule (Iba-1). Oxidative stress was assessed using 8-hydroxydeoxyguanosine (8-OHdG). The presence and distribution of melatonin receptors (MT1, and MT2) in the brain was also assessed. The areas of interest assessed were the subventricular zone (SVZ), periventricular white matter (PVWM), subcortical white matter (SCWM), intragyral white matter (IGWM), corpus callosum (CC), external capsule (EC) and cortex (Cx) layers; these were examined in the fetal sheep brain at the level of the striatum.

In single-label immunohistochemistry, sections were heated with citric acid buffer for 3 × 5 min followed by incubation in 0.3% hydrogen peroxide to remove endogenous peroxidase. Sections were then incubated in blocking solution (phosphate-buffered saline, PBS; 0.1 m, pH 7.4), 5% normal goat serum for 1 h at room temperature to block nonspecific binding, followed by incubation with primary antibody. Oligodendrocyte lineage cells were identified using primary rabbit polyclonal Olig-2 antibody (1:1000; Merck Millipore Corporation, Bayswater, VIC, Australia) diluted in DAKO antibody diluent, where olig2 is a specific oligodendrocyte transcription factor essential for all oligodendrocyte lineage cells. Primary mouse CNPase (1:300; Sigma-Aldrich, St. Louis, MO) was diluted in 1% bovine serum albumin 0.1% tween-PBS solution and primary rat MBP (1:500, Merck & Co, Rahway, NJ) was diluted in 10% normal goat serum (NGS) in PBS. Mouse Anti-NeuN (1:100; Millipore Corporation, Billerica, MA) antibody diluted in 2% NGS and 1% BSA PBS was used to identify neuronal cells. GFAP (1:400; Sigma-Aldrich, St. Louis, MO) was diluted in 0.3% TX-PBS. Microglia were identified using rabbit anti-Iba-1 antibody (Wako Pure Chemical Industries Ltd., Osaka, Japan) diluted 1:1000 in 0.2% TX-PBS solution and apoptotic cells were identified using rabbit polyclonal anti-caspase-3 antibody (1:1000; R & D systems, Minneapolis, MN). Oxidative stress was studied using 8-OHdG (1:200, JaICA, Japan). Melatonin receptors (MT-1, MT-2) were studied using MTR1A (1:100, Abbiotec, CA) and MTR1B (1:100, Bioss Antibodies, MA) antibodies. All primary antibodies were incubated at 4 °C overnight.

All sections were treated with a secondary antibody (1:200; biotinylated anti-rabbit, anti-rat or anti-mouse IgG antibody; Vector Laboratories, Burlingame, CA) for 1 h at room temperature stained with 3,3′-diaminobenzidine (DAB; Pierce Biotechnology, Rockford, IL). Sections were counter-stained with either haematoxylin (MBP single label imuunohistochemistry) or nuclear fast red (Olig-2 and CNPase single label imuunohistochemistry). Positive and negative control sections were included in each run. Two adjacent sections from each fetal brain were examined.

### Quantitative analysis of brain injury

Sections were viewed at a magnification of ×40 using light microscopy (Olympus BX-41, Japan) and examined in coded slides to ensure the assessors (AM, AKAAR) were blinded to the groups. Immunoreactive cell counts and/or density of stain were counted in three fields of view within a given region on two slides per animal to give six fields of view per region per animal, which were then averaged per animal. The number of Olig-2-immunopositive cells per region were calculated by converting the image to eight-bit resolution and then calculating a threshold level for cell detection using a digital image analysis program (ImageJ v1.47, NIH, Bethesda, MA). NeuN neuronal positive cells were quantified in two cortical layers: mid cortical and deep cortical layer. *Healthy* NeuN-neurons were manually counted; cells with a normal nucleus, a clearly defined nuclear membrane and light cytoplasmic staining were counted as healthy neurons, while cells with condensed nuclei, and abnormal cytoplasmic morphology (with or without intracellular vacuoles) were labelled as unhealthy. Manual cell counting was performed for GFAP-positive cells, activated or amoeboid microglia cells, caspase-3, MT-1 and MT-2-positive cells per field of view, using Image J. Positive staining for the MT1 receptor was found only in the cortical layers (bins 2–4). We utilised threshold-based digital analysis to quantify the density of CNPase, MBP and MT2 immunostaining (Image J, Bethesda, MA).

### Statistical analysis

Statistical comparisons and graphs were prepared using GraphPad Prism (GraphPad Software v9, San Diego, CA). Data are presented as the mean ± standard error of the mean (S.E.M.). Main effects analysis for growth status (FGR vs. control) and melatonin (treated vs. non-treated) were analysed using two-way ANOVA for body and organ weights, and brain histopathology for each brain region of interest to assess cell counts and density of staining. Additionally, within each brain region we used a one-way ANOVA for direct comparisons amongst the four study groups. Blood gas data were analysed by two-way repeated measures ANOVA. Significance was accepted at *p* < 0.05.

## Results

### Animal wellbeing

Fetal arterial blood samples were collected for 10 days after SUAL surgery for assessment of pH, oxygen saturation (SaO_2_), partial pressure of oxygen (PaO_2_), and partial pressure of carbon dioxide (PaCO_2_). Two-way ANOVA showed that there were no significant changes over the period of study for parameters examined (*p* > 0.05). On D10 post SUAL, all parameters were within the normal range in control fetuses; pH 7.33 ± 0.00, PaO_2_ 28.3 ± 3.6 mmHg, PaCO_2_ 47.2 ± 3.0 mmHg, SaO_2_ 78.6 ± 8.5%. FGR fetuses were hypoxic on D10 (PaO_2_ 20.2 ± 3.05 mmHg and SaO_2_ 60.8 ± 7.28%; *p* < 0.05). There were no other significant differences between groups and melatonin administration did not alter blood gas parameters.

While fetuses in the FGR group weighed less than those in the control group (2.51 ± 0.13 vs. 2.73 ± 0.14 kg), overall the two-way ANOVA showed no significant differences between groups, *p* = 0.18, including for melatonin treatment (FGR + MLT 2.18 ± 0.12 kg vs. control+MLT 2.54 ± 0.24 kg).

### Neuropathology

#### White matter development

**Fig. 1:**
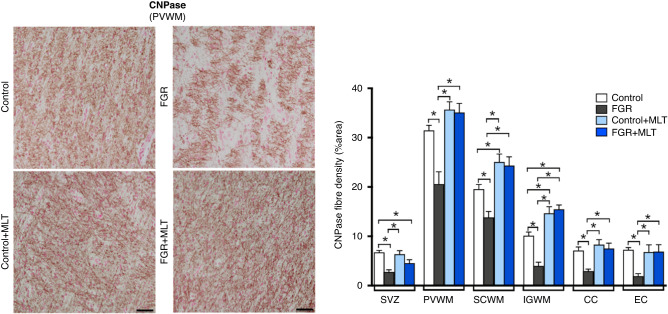
Left panel: representative photomicrographs of CNPase fibre density staining in PVWM of lamb groups. Scale bar = 100 μm. Right panel: Two-way ANOVA analysis showed the following significant results: SVZ: FGR: <0.0001; PVWM: Interaction: 0.004, FGR: 0.001, MLT: <0.0001; SCWM: FGR: 0.03, MLT: <0.0001; IGWM: Interaction: 0.001, FGR: 0.01, MLT: <0.0001; CC: FGR: 0.01, MLT: 0.004; EC: Interaction: 0.001, FGR: 0.002, MLT: 0.008. *Denotes significant differences (*p* < 0.05) on one-way ANOVA analysis between marked groups in the specific brain region.

**Fig. 2:**
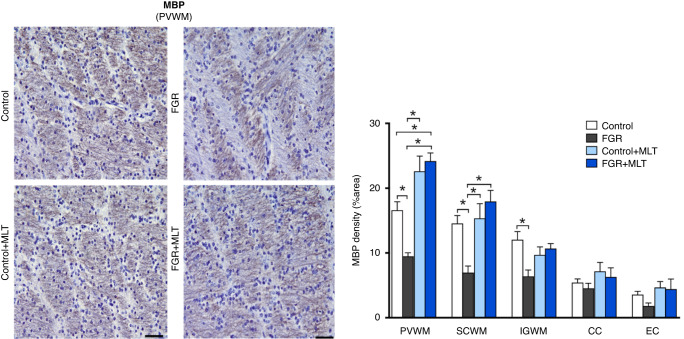
Left panel: representative photomicrographs of MBP density staining in PVWM of lamb groups. Scale bar = 100 μm. Right panel: Two-way ANOVA analysis showed the following significant results: PVWM: Interaction: 0.01, MLT: <0.0001; SCWM: Interaction: 0.005, MLT: 0.001; IGWM: Interaction: 0.01. *Denotes significant differences (*p* < 0.05) on one-way ANOVA analysis between marked groups in the specific brain region.

Olig-2, MBP, and CNPase-positive immunostaining were quantified in white matter brain areas. Oligodendrocyte lineage (Olig2+) cell counts did not differ across groups for any brain region examined (Supplementary Fig. 1). The immature/mature myelin protein CNPase (Fig. 1) showed a consistent pattern of change across brain regions and experimental groups: Two-way ANOVA showed that CNPase+ density was reduced in FGR brains compared to control brains within the subventricular zone (SVZ, *p* < 0.0001), periventricular white matter (PVWM, *p* = 0.001), subcortical white matter (SCWM, *p* = 0.03), intragyral white matter (IGWM, *p* = 0.01), corpus callosum (CC, *p* = 0.01), and external capsule (EC, *p* = 0.002). Melatonin significantly improved CNPase+ density within the PVWM (*p* < 0.0001), SCWM (*p* < 0.0001), IGWM (*p* < 0.0001), CC (*p* = 0.004) and EC (*p* = 0.008). Within brain region differences were then assessed across groups, with significant differences shown in Fig. 1, demonstrating that CNPase density was improved in FGR + MLT groups compared to FGR alone within the PVWM, SCWM, IGWM, CC, and EC (all *p* < 0.05), but also that control+MLT was increase above control within the SCWM and IGWM (both *p* < 0.05). While the mature myelin protein MBP (Fig. 2) showed a similar profile, the two-way ANOVA did not find a main effect of FGR across any brain region studied, while melatonin increased MBP staining in the PVWM (*p* < 0.0001) and the SCWM (*p* = 0.001). Within brain regions of interest, FGR brains demonstrated reduced MBP staining density within the PVWM, SCWM and IGWM (all *p* < 0.05), FGR + MLT was increased compared to FGR in the PVWM and SCWM (both *p* < 0.05), but MBP density was not different in control+MLT compared to the control untreated group.

#### Neuronal cell count

**Fig. 3:**
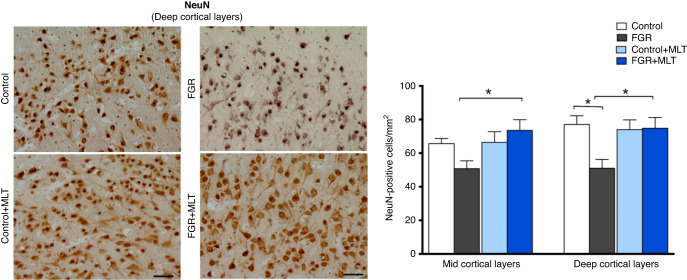
Left panel: representative photomicrographs of NeuN staining in deep cortical layers of lamb groups. Scale bar = 100 μm. Right panel: Two-way ANOVA analysis showed the following significant results: mid cortical layers: Interaction: 0.0243, MLT: 0.0166, FGR: 0.4134; deep cortical layers: Interaction: 0.0197, MLT: 0.0675, FGR: 0.0278. *Denotes significant differences (*p* < 0.05) on one-way ANOVA analysis between marked groups in the specific brain region.

We next quantified neurons (NeuN+ cells) in the mid and deep layers of the cortex (Fig. 3). At the mid-cortical layer, two-way ANOVA found no overall main effect of FGR (*p* = 0.413), but an increase in NeuN+ cell counts with melatonin treatment (*p* = 0.016). Within-group analysis showed a significant increase in cell counts in the FGR + MLT mid-cortex compared to FGR alone (*p* < 0.05). At the level of the deep cortex, two-way ANOVA found that neuron cell counts were decreased in FGR groups (*p* = 0.028), but no significant effect of melatonin treatment (*p* = 0.067). Within-group analysis of the deep cortical layer showed a significant decrease in FGR neuron cell counts compared to control, and FGR + MLT cell counts were increased compared to FGR alone (both *p* < 0.05).

#### Neuroinflammation

**Fig. 4:**
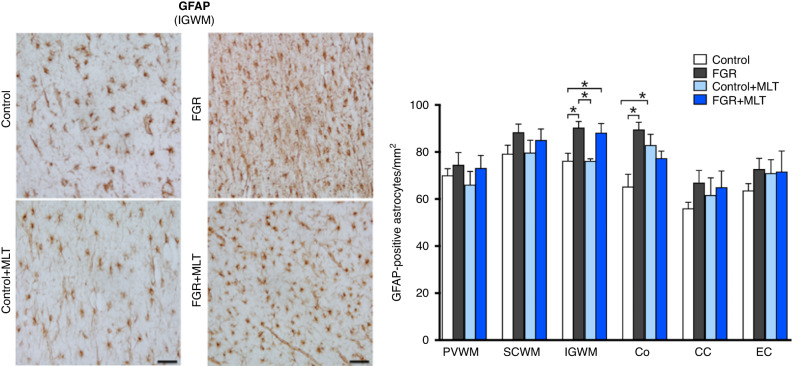
Left panel: representative photomicrographs of GFAP staining in IGWM of lamb groups. Scale bar = 100 μm. Right panel: Two-way ANOVA analysis showed the following significant results: IGWM: FGR: 0.0004; Co: Interaction: 0.0043. *Denotes significant differences (*p* < 0.05) on one-way ANOVA analysis between marked groups in the specific brain region.

**Fig. 5:**
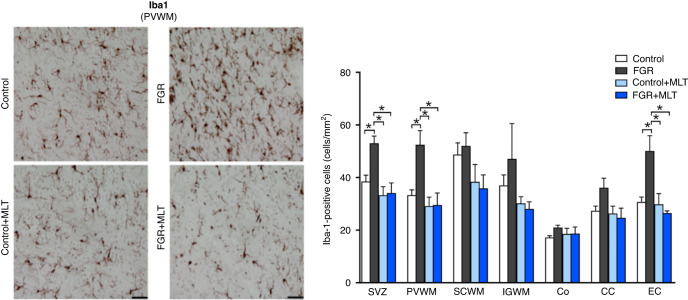
Left panel: representative photomicrographs of Iba-1 staining in PVWM of lamb groups. Scale bar = 100 μm. Right panel: Two-way ANOVA analysis showed the following significant results: SVZ: Interaction: 0.03, FGR: 0.02, MLT: 0.007; PVWM: Interaction: 0.01, FGR: 0.01, MLT: 0.001; CC: MLT: 0.04; EC: Interaction: 0.002, FGR: 0.02, MLT: 0.001. *Denotes significant differences (*p* < 0.05) on one-way ANOVA analysis between marked groups in the specific brain region.

We assessed the neuroinflammatory status of the brain, via assessment of astrocyte cell count (GFAP+ cells) and microglia cell count (Iba-1+ cells), as shown in Figs. 4 and 5, respectively. Two-way ANOVA of GFAP+ astrocytes/mm^2^ across different brain regions demonstrated that FGR was associated with an increase in astrocytes within the IGWM only (*p* = 0.0004), and with no overall effect of melatonin treatment. Within-group effects showed that indeed FGR was increased compared to control in the IGWM and the cortex (both *p* < 0.05), but FGR + MLT was not different to FGR alone (Fig. 4). Two-way ANOVA of Iba-1+ microglia (Fig. 5) showed more consistently that FGR was associated with an inflammatory state, with increased microglia in the SVZ (*p* = 0.02), PVWM (*p* = 0.01) and EC (*p* = 0.02) brain regions, while melatonin reduced microglia within the SVZ (*p* = 0.007), PVWM (*p* = 0.001), CC (*p* = 0.04) and the EC (*p* = 0.001). Within-group analysis across brain regions confirmed that FGR was predominantly associated with increased microglia cell counts, compared to the control group, within the SVZ, PVWM and EC (all *p* < 0.05) and FGR + MLT brains demonstrated a decreased microglia cell count compared to FGR alone within the SVZ, PVWM, and the EC (all *p* < 0.05).

#### Oxidative stress

Oxidative stress in the brain was studied using 8-OHdG. No significant differences were observed in 8-OHdG across the four animal groups (Supplementary Fig. 2).

#### Melatonin receptors

**Fig. 6:**
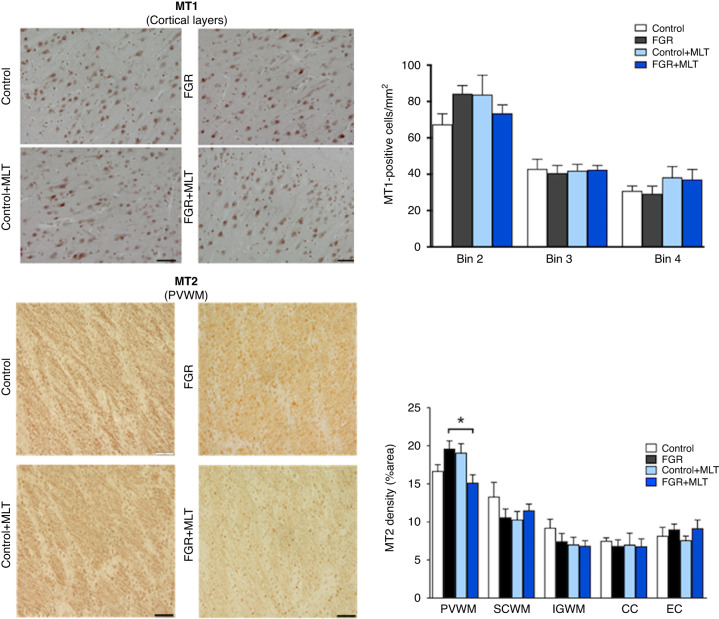
Top figure: left panel: representative photomicrographs of MT1 receptor staining in cortical brain regions (bins 2–4) showing no difference between lamb groups. Scale bar = 100 μm. Right panel: two-way ANOVA analysis showed no significant results. No interactions or significant differences between groups seen. Bottom figure: left panel: representative photomicrographs of MT2 receptor staining in PVWM of lamb groups. Scale bar = 100 μm. Right panel: two-way ANOVA analysis showed no significant results between groups. *Denotes significant differences (*p* < 0.05) on one-way ANOVA analysis between marked groups in the specific brain region.

Finally, melatonin receptors (MT1 and MT2) were assessed across our brain regions of interest. We observed staining of these receptors in cells with visible nuclear membrane and light cytoplasmic staining, and in cells with non-evident nuclei, only with staining in the cytoplasm. We quantified these cell staining patterns separately and performed manual counts. MT1 was predominantly localised to grey matter-rich regions and therefore we performed quantification in the cortex (Fig. 6: top figure). Conversely, MT2 receptors were predominantly localised to white matter regions and therefore we performed quantification in the PVWM, SCWM, IGWM, CC, and EC. No significant differences were observed in the MT1 receptor-positive cells in cortical regions. Two-way ANOVA showed no significant interactions or effects of FGR or MLT on MT2 receptor expression, but MT2 receptor density was increased in PVWM in the FGR group, and within groups analysis showed a reduction in the FGR + MLT group compared to FGR (*p* < 0.05) (Fig. 6: bottom figure).

## Discussion

Early-onset placental insufficiency and FGR presents an obstetric challenge, due to the high likelihood of mortality and morbidity^5^ and high incidence of neuropathology.^7^ Early-onset FGR is, however, detectable in utero via measures of poor growth and placental insufficiency,^4,5^ therefore providing an opportunity for antenatal therapeutic intervention that tends not to be available for late-onset FGR. In the current study, we used an early-onset placental insufficiency and FGR fetal sheep model to evaluate the neuroprotective effects of maternal melatonin administration. In this study, we provide striking evidence that maternal melatonin therapy improves myelination and prevents the cortical neuronal degeneration that was apparent in the FGR cohort of animals. The most likely neuroprotective mechanism of action of melatonin treatment is via its anti-inflammatory actions, as we did not find evidence of elevated levels of oxidative stress in FGR brains in this experimental series. Within the developing fetal sheep brain, we present novel evidence for the presence of the melatonin receptors MT1 and 2, predominantly localised within the grey matter and white matter, respectively. This data provides the first preclinical evidence that melatonin is an effective neuroprotective therapy for early-onset placental insufficiency and FGR.

Our results confirm the presence of placental insufficiency, induced via the surgical process of single umbilical artery ligation at 0.6 gestation, with our FGR cohort significantly hypoxaemic at day 10 after surgery. This is in contrast to the late-onset FGR model (0.7 gestation) that we have examined previously which shows an acute hypoxaemia soon after surgery.^8^ That this preclinical study produces chronic hypoxia in fetal sheep is important as it confirms placental dysfunction, and hypoxia is the chief pathophysiological mechanism implicated in FGR and its related morbidities in the human fetus and newborn.^3^ Interestingly, while the FGR cohort tended to weigh less than the control animals (~8%), this difference was not significant. This is at odds with what we have shown previously for early-onset FGR in fetal sheep (~20% decrease weight compared to control),^8^ but appears to reflect that in the current study, our control group lambs were slightly smaller than expected. We did not find that melatonin administration had any effect on fetal blood gas parameters, suggesting an absence of direct effect (or adverse effect) of melatonin on fetal or placental blood flow or oxygen consumption. Nor did we observe that maternal melatonin administration had any effect on fetal body weight. A previous study in late-onset FGR fetal sheep showed that maternal melatonin administration improved both fetal oxygenation status and weight gain,^24^ and a recent study of maternal melatonin implants at varying doses in sheep showed an improvement of growth (fetal weight) at term gestation but this was limited to an effect in male offspring.^25^ Overall, pre-clinical evidence for maternal melatonin administration and its effect on fetal growth is divided. In a developmental hypoxia model of growth restriction at high altitude, antenatal melatonin had a negative effect on growth.^26^ On the other hand, multiple experimental studies in growth restricted rats have shown a beneficial effect of maternal melatonin on fetal growth.^27–29^ The effects of antenatal melatonin on different animal species, at different doses and assessments of fetal sex needs to be further explored. Two small human trials in which melatonin has been given maternally during pregnancy for preeclampsia^13^ and FGR^19^ also report mixed results, with the Hobson study finding an increased incidence of growth restriction in infants exposed to melatonin, and the Miller study finding no difference in birth weights with or without antenatal melatonin. There is currently a placebo-controlled randomised clinical trial to examine the neurodevelopmental outcomes of FGR infants treated with either melatonin or placebo (The Protect-Me trial) during pregnancy that is currently recruiting (*n* = 336),^30^ and we keenly await these results.

In the current study, the principal brain pathology observed in response to placental insufficiency and FGR was diffuse white matter injury. This was evident with deficits in two principal myelin proteins, CNPase and MBP, which showed reduced density across multiple brain regions including the PVWM, SCWM, IGWM areas. This was not accompanied by a loss of total oligodendrocyte lineage cells (Olig2-positive). Myelin is a multilamellar membrane, predominantly comprising lipids (~80%), followed by myelin proteins, with this lipid-rich myelin produced by mature oligodendrocytes. Myelination within the developing brain is essential for establishing connectivity and facilitating rapid action potential transduction along axons. Results presented here reiterate multiple preclinical studies that demonstrate hypomyelination in response to placental insufficiency and fetal hypoxia.^8,31^ These results also provide a histopathological basis for the white matter deficits that underlie MRI observations in clinical studies of human infants with early-onset FGR, including a significant reduction in myelination^32^ and microstructural alterations in multiple white matter tracts.^33^ White matter injury in human infants and children is positively correlated with neurofunctional impairments, including the motor disability cerebral palsy, and thus strategies to improve white matter development are highly sought after.^34^

The most profound effects observed with antenatal melatonin administration were the positive effects on myelination within the fetal brain. Antenatal melatonin concentration significantly improved myelin density, as shown by an increase in both CNPase and MBP. The mechanism of this improvement in myelin content in this experiment is intriguing. As there was no significant impact on total oligodendrocyte number, it is possible that this effect on myelination was inflammation mediated. Neuroinflammation and white matter injury often go hand-in-hand, observed in preclinical^8,35^ and clinical studies.^36^ We are also mindful that melatonin administration promoted increased levels of CNPase protein over and above control levels in the subcortical and intragyral white matter regions, and MBP protein in the periventricular white matter. There are two things that should be considered in this finding, (i) it is unlikely that melatonin administration would be given in a healthy control pregnancy (note some growth restricted fetuses may be constitutionally small), and (ii) it is not known whether this enhanced myelination is due to the laying down of extra myelin, or promotion of normal maturational events. We suggest that the second point should be explored further in longer-term preclinical experiments.

In the current study, we observed a strong microglial response in the FGR brains, localised to the SVZ, PVWM, and EC, while astrogliosis was present in IGWM and the cortex. Maternal melatonin administration was strongly anti-inflammatory, with all microglial cell counts normalised across all regions in the FGR + MLT animals. In contrast, melatonin exposure did not prevent astrogliosis. This result provides evidence that white matter injury is more closely associated with microglial cell proliferation and activation, than astrogliosis, but that melatonin is strongly protective for white matter within the FGR brain via its anti-inflammatory actions. Melatonin has been noted to have anti-inflammatory actions in pre-clinical models of both fetal and neonatal conditions.^16,37^ Mitigation of inflammation induced brain damage may be co-mediated via suppression of oxidative stress pathways, and melatonin receptors.^38,39^

Melatonin administration also had a modest but evident effect on neuronal cell wellbeing in the cortex of FGR brains. We counted only ‘healthy’ NeuN-positive neurons and found a reduced number of healthy neurons in the deep cortex of FGR brains. Excitingly, melatonin treatment was associated with improved healthy neuronal cell counts. It is not immediately apparent why the neurons demonstrate improved health with melatonin exposure, given that the cortex of these animals did not show profound microglial activation, astrogliosis, or oxidative stress. There are however other factors that are disrupted in the FGR brain that may play a part in this neuroprotective effect. For example, we have previously shown that cortical grey matter blood flow in the FGR fetal sheep brain is less than half compared to control fetuses,^40^ and others have shown that melatonin actively vasodilates and thus normalises cerebral circulation in chronically hypoxic neonatal sheep.^41^ The neurovascular unit within the FGR brain is also less robust, due to end-feet of astrocytes becoming detached from the vascular wall,^42^ and melatonin is shown to promote this astrocytic attachment.^17^ Finally, preclinical studies have also shown that brain-derived neurotrophic factor (BDNF) is significantly reduced in grey matter regions of growth restricted guinea-pig brains,^43^ where BDNF is critical to support both normal neuronal processes and development in early life and beyond. BDNF is also an essential regulator of dendritic outgrowth in fetal and neonatal life and therefore future studies should assessor whether FGR and melatonin differentially affect dendritogenesis during pregnancy.

Melatonin has both receptor-mediated and receptor-independent functions, thus accounting for its diversity of neuroprotective actions.^44^ We assessed for the presence and localisation of the MT1 and 2 receptors within the fetal sheep brain, and to determine whether they were differentially regulated by either FGR or antenatal melatonin administration. We did not see a significant change in melatonin receptor numbers in the fetal brains, irrespective of FGR status or maternal melatonin administration, except in one brain region (MT2, PVWM). Multiple studies have demonstrated that melatonin has a neuroprotective capacity either in pregnancy or with administration to the neonate after birth,^12,14,16,19^ and MT receptors are present in the fetal brain.^45^ Melatonin receptors are also present in the placenta and were noted to be reduced in the placenta of pregnancies affected by FGR in a recent study.^46^ This indicates that even if circulatory melatonin levels in pregnancy are normal, the actions of melatonin may be subdued in FGR pregnancies. While the anti-inflammatory effects of melatonin appear to be principally mediated by melatonin receptors, its antioxidant actions are predominantly receptor-independent.^47^ In a rat model of white matter damage, melatonin administration was associated with promoting oligodendrocyte maturation. This action was possibly melatonin receptor mediated and reproducible in vitro.^48^ The observation in the current study that there were not pronounced changes in melatonin receptor distribution or density in the developing brain in response to either FGR or exogenous melatonin administration provides reassurance that the receptor-mediated actions of melatonin (especially its anti-inflammatory role) were not adversely affected.

This established model of early-onset FGR lends itself to good examination for effects of FGR and experimental therapeutics. Unfortunately, animal models and experiments have their limitations. As fetal lamb gestation was terminated at a preterm age (~127 days of a full-term lamb gestation of 148 days), long-term effects of FGR and melatonin administration could not be systematically assessed in this study. Notably, we did not see a significant reduction of lamb body weights in this experiment, although we can confirm from our arterial blood samples that placental insufficiency caused fetal hypoxaemia. It is important to note that while we had relative hypoxaemia in the FGR, the range of PaO_2_ was not as low as previously reported in FGR lambs.^49^ While a number of brain regions were assessed, a number of other brain regions that may be affected in FGR, were not evaluated. These include the hippocampus and cerebellum, which have been shown to be affected in FGR.^50^ It was unexpected that we did not find evidence of oxidative stress in response to placental insufficiency; we quantified cellular evidence of the protein 8-hydroxy-2’-deoxyguanosine (8-OHdG), which is a marker of DNA oxidative damage. Previous work by us and others have shown an upregulation of cerebral oxidative stress in FGR offspring using end-stage markers for lipid peroxidation, such as 4-hydroxynonenal and malondialdehyde,^51^ and consideration should be given to the variability of these outcomes using these assessments, together with the specific effects of melatonin.

## Conclusions

Maternal melatonin administration in a lamb model of early-onset FGR leads to increased myelination of white matter brain regions in the fetus, possibly mediated by decreased inflammation. This is likely to have neuroprotective effects in fetuses of compromised pregnancies leading to improved neurodevelopmental outcomes, and warrants further preclinical and clinical evaluation.

### Supplementary information

Supplementary Figures

## Data Availability

The data that support the findings of this study are available from the corresponding author upon reasonable request.
