# Supplementary material for: Neuroprotective effects of maternal melatonin administration in early-onset placental insufficiency and fetal growth restriction
Source: Pediatr Res. 2024 Jan 15;95(6):1510–8. doi: 10.1038/s41390-024-03027-4 (PMC11126390; doi:10.1038/s41390-024-03027-4)
Supplement: Supplementary file 1 — Supplementary Figures [file 41390_2024_3027_MOESM1_ESM.pdf]

Supplementary Figure 1

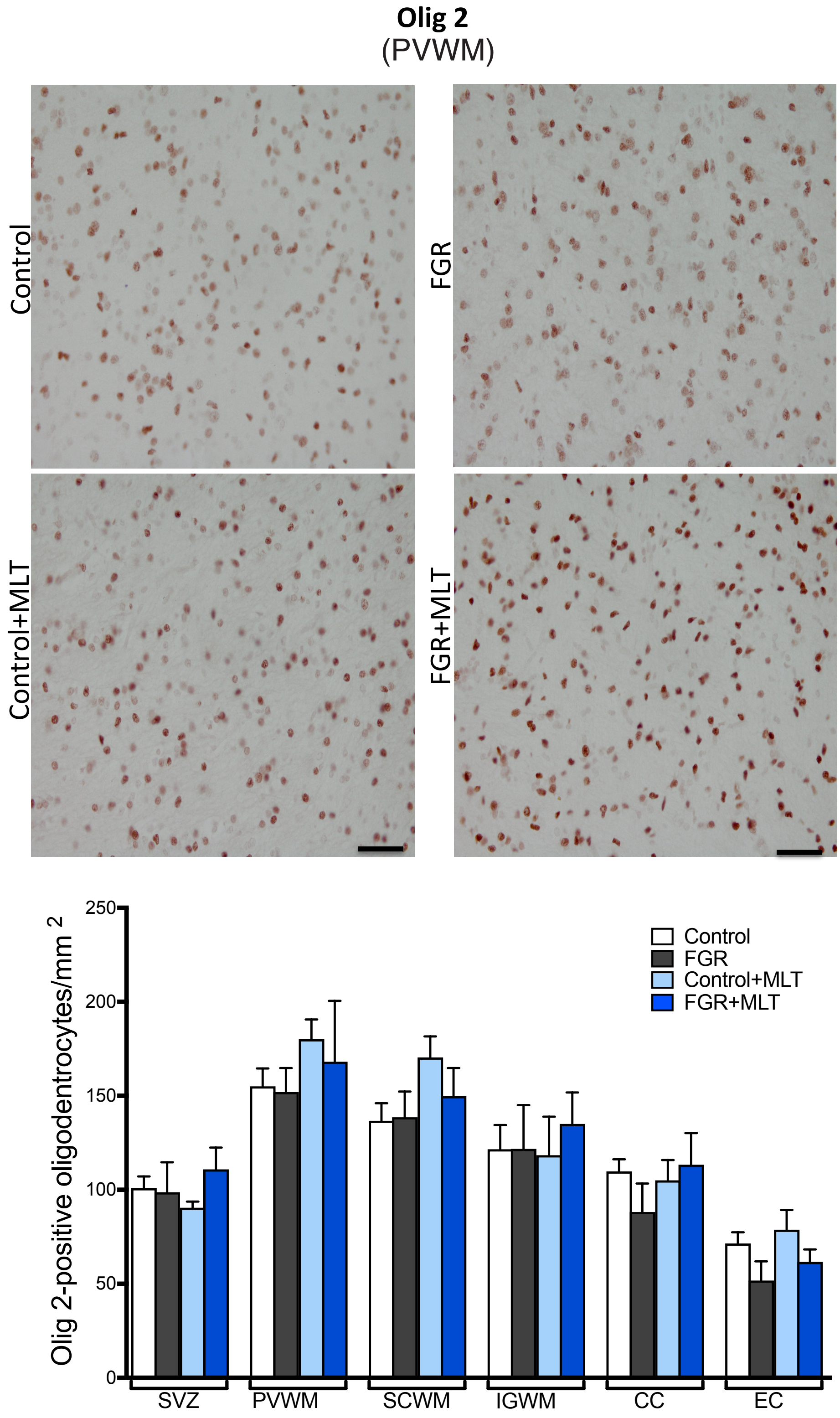

Top panel: Representative photomicrographs of Olig 2 immunohistochemistry stain in PVWM brain region showing no difference between lamb groups. Scale bar= 100um. **Bottom panel:** Analysis of Olig 2 positive cells across different brain regions. No interactions or significant differences between groups seen.

Supplementary Figure 2

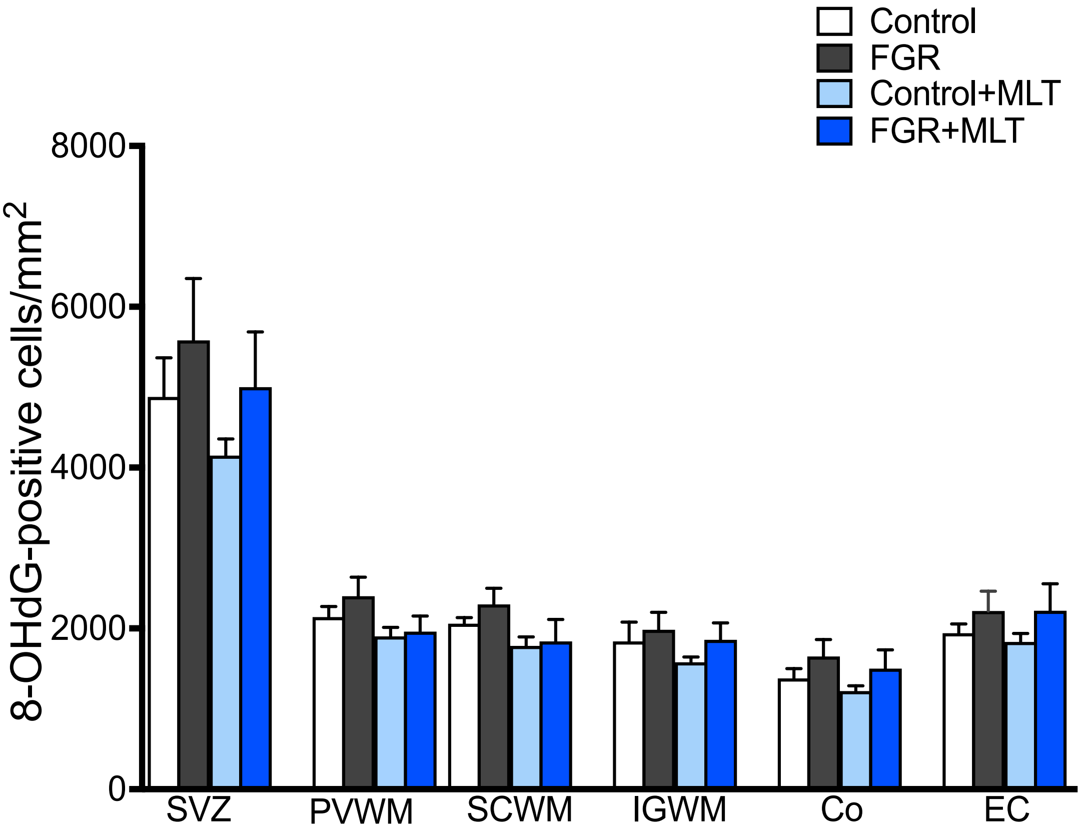

Analysis of 8-OHdG positive cells across different brain regions. No interactions or significant differences between groups seen.
